# An auto-ethnographic reflection on the nature of nursing in the UK during the Covid-19 pandemic

**DOI:** 10.1177/13634593211064122

**Published:** 2021-12-11

**Authors:** Helen T Allan

**Affiliations:** Middlesex University, UK

**Keywords:** autoethnography, commodification of nursing care, COVID-19, distal patient, isolation nursing

## Abstract

In this article I discuss the effects on the patient experience of isolation nursing during the CoronaVirus Disease (COVID)-19 pandemic. An unintended consequence of isolation nursing has been to distance patients from nurses and emphasise the technical side of nursing while at the same time reducing the relational or affective potential of nursing. Such distanced forms of nursing normalise the distal patient in hospital. I consider ways in which this new form of distanced nursing has unwittingly contributed to the continued commodification of nursing care in the British NHS. Autoethnography is used to describe and reflect on the illness experience, the experiences of caregivers and the sociocultural organisation of health care. The findings discuss three areas of the illness experience: intimate nursing care; communication; the ‘distanced’ patient experience.

## Introduction

Global CoronaVirus Disease (COVID)-19 has profoundly affected all frontline workers, especially nurses. Hospitalisation and death rates have increased the physical and emotional workload for front-line healthcare staff (Fernandez et al., 2020) who have also been at risk of infection and death from COVID-19 (Nguyen et al., 2020; Wise, 2021). The effects of the pandemic will be felt for many years to come (MacGuire, 2020).

In many countries patients have been cared for in isolation and with personal protective equipment (PPE) to reduce the spread of infection (Yoo et al., 2020). Family or close friends have been unable to visit patients in hospital in order to reduce cross-contamination (Landers et al., 2010). The lack of family involvement has been particularly distressing (Downar and Kekewich, 2021) as isolation nursing has been shown to effect patient well-being (Barratt et al., 2010; Purssell et al., 2020).

In this article, I use autoethnography to explore the negative effects of the use of PPE and isolation nursing on the patient experience. I suggest that an unintended consequence of isolation nursing has been to reinforce the more technical side of nursing as nurses’ relationships with patients have necessarily become more distanced. I argue that such distanced forms of nursing (May et al., 2001, 2006), where subjectivity and emotions are excluded from nurse-patient relationships, have normalised the distal patient in hospital. Such forms of nursing are evidence of the continued commodification of British nursing.

## Background

In 1953, Goddard argued that nursing could be divided into technical, affective (relational) and basic work (bedside or intimate care). She argued that nurses valued technical care more highly over intimate or emotional care of patients and generally avoided relational care over technical tasks. Menzies (1970) argued that technical tasks reduce emotional intimacy and that to reduce the anxiety of emotional intimacy, nursing tasks (at least in the UK) were allocated according to the hierarchy of value observed by Goddard (1953). Over time, lesser value tasks, including bedside or intimate care, were delegated by registered nurses to unregistered healthcare assistants (HCAs) (Allan, 2017; Allan et al., 2018). This hierarchy of value related to nursing tasks held except for a period in the 1990s, in what was known as the ‘new nursing’ in the UK (Savage, 1997) when patient-centred nursing was promoted.

The avoidance of relational care in nursing was shaped by the organisational structures in which nursing took place in the second half of the 20th century (Dashtipour et al., 2021; Fotaki and Hyde, 2015; Tutton and Langstaff, 2015). After 2000, the emergence of new public management (NPM) across many developed countries’ health care systems (Pedersen and Roelsgaard Obling, 2019) increased the organisational tendency of nursing to avoid intimacy with patients, and facilitated the commodification of nursing care (Martínez-Rodríguez et al., 2020; Smith and Mackintosh, 2007). Commodification of nursing care defines nursing activities as measurable outputs which have economic value within new frameworks of health care organisation (Martínez-Rodríguez et al., 2020). Underpinning these frameworks were neo-liberal policies applied to healthcare which reshaped nurses’ relationships with patients through the restructuring of the organisation of care. This was usually through the reintroduction of task allocation instead of patient allocation (Allan et al., 2008); and the privatisation of care tasks (Smith and Mackintosh, 2007). As a result of NPM, intimate ‘unskilled’ caring for patients and Goddard’s affective and basic tasks, were delegated to unregistered nursing assistants (Martínez-Rodríguez et al., 2020) thus reinforcing the low value assigned to the task by the profession. This was mirrored across developed health care systems. Thus, since 2000 under NPM, delivery of intimate care as a core duty of British nursing has gradually disappeared (Allan, 2018; Johnson et al., 2015) although not without resistance (Allan, 2018).

NPM in the UK, under both Conservative and Labour governments, has led to a fragmented NHS (Peacock, 2019) with: (i) a failed internal ‘market’ model, (ii) commissioning devolved to local level which is anything but local, (iii) layers of bureaucracy which are intended to keep the Department of Health at arms-length from any failures in the NHS, (iv) an incoherent workforce and education/training strategy which has relied on overseas recruitment while nursing vacancies run a 40%. Such poor workforce planning has led to occupational stress where 40% of newly qualified nurses leave within 2 years of qualifying (Open University, 2018). These systemic problems suggest a health care service which fails its nurses and makes it more difficult for them to care for patients (O’Driscoll et al., 2018; Smith, 2013).

The Francis Report (Francis, 2013) into nursing care in hospitals in Mid Staffordshire revealed the risks of the delegation of intimate (basic) and affective care to untrained HCAs. Due to the focus on NPM, that is on nursing tasks rather than relational care, Francis (2013) reported a disregard of relational care across the hospitals from all staff. The absence of relational nursing care led (alongside other structural factors) to deficits in care of patients. Francis concluded that relational care was essential to all heath care tasks, not only nursing care. There was much debate over how these deficits in care had been allowed to happen (Bradshaw, 2017; Fotaki and Hyde, 2015; Paley, 2014). It became apparent that poor staffing, low morale, poor leadership and a return to tasks rather than holistic person-centred care had also been influential in shaping the outcomes in Mid Staffordshire hospitals (O’Driscoll et al., 2018; Traynor, 2014).

I argue here that the new forms of distal nursing during the pandemic are evidence of the continued commodification of nursing care in the British health service (Dashtipour et al., 2021; Hyde and Davies, 2004). They build on existing trends in nursing to devalue the subjective in nursing while at the same time, upholding and reinforcing the values underpinning NPM.

## Theoretical framework

May et al. (2001) argue that therapeutic relationships nurture relations between patients and their carers (doctors, nurses, assistant workers, therapists). Any changes to how care is delivered, in tele-medicine or tele-fertility care for example, interfere with such relationships and the delivery of relational care. Distal forms of health care delivery produce and reproduce new discourses about the nature and process of health care along with a different valuing of different forms of delivery. In the British NHS, the value of relational care, irrespective of who delivers that care, has been underestimated (Allan, 2002, 2009) while at the same time relational, compassionate care has been idealised in professional discourses and government policy (Dashtipour et al., 2021; O’Driscoll et al., 2018). May et al. (2001) show how therapeutic interactions and their potential effects are threatened by the changes telemedicine has on clinical interactions; these changes occur in two forms: as hard, material artefacts (video, telephones, computers) and as soft technology such as the forms of communication shaped through the use of the artefacts. I suggest that PPE might be considered an artefact and the reliance on exaggerated modes of communicating such as gestures, facial expressions, proxemics (use of space in communication), body language, touch and intensity of gaze, are a new form of communication driven by the introduction of PPE (Senicola et al., 2020).

In a later article exploring the use of telepsychiatry, May et al. (2006) describe a new type of patient, the distal patient, who is constructed through new forms of e-health technology. These technologies are used to ‘solve’ the problem of the patient’s subjectivity which is seen to interfere with objective history taking and diagnosis. May et al. (2006) argue that a distance is created between service provider and user: ‘non-human actors can be incorporated into the clinical encounter to decouple experience and management’ (p. 1028; that is, to extract the subjective data in objective form to aid diagnosis and management of clinical conditions. The intention of these encounters is to ‘extract from the life-world only [those] data that [are] necessary to determine the trajectory of a chronic illness’ (May et al., 2006: 1028). It is worth noting, although not the focus of this paper, that telemedicine used during the pandemic, although well intentioned to prevent the spread of COVID-19, has possibly aided in creating distance between patient and provider. In this article I focus on the distal patient in the hospital setting and the loss of subjectivity from the forms of nursing which have emerged during the pandemic.

## Methodology and methods

Knowing a culture, even one with which we are intimately engaged, has been described as a never-ending story (Crumbie, 2005: 77). Autoethnography is a methodology in which the process of knowing one’s own culture becomes the purpose of the research; an autoethnographer becomes the research instrument in his/her own culture. In this article, as part of my ‘never-ending’ story of being a nurse, I draw on personal experiences as a patient with COVID-19 as well as reflections on those experiences which are shaped by over 40 years of being a nurse and more recent practice as a nurse researcher. Autoethnography is a qualitative research methodology which draws on a qualitative researcher’s use of interpretive practices (Denzin and Lincoln, 2000). Central to autoethnography is reflexivity which allows for the interpretation of the personal or autobiographical experiences of the researcher through the broader sociocultural context (Foster et al., 2006; Pariseau-Legault, 2018). Foster et al. (2006) argues that in autoethnography, the ‘self’ is seen as continually connected to the social world, as being constantly in flux, thus creating multiple interpretations (Gadow, 2000).

Using autoethnography (Pariseau-Legault, 2018; Peterson, 2015), I discuss changes in hospital nursing work in the British context brought about by COVID-19 and the unintended development of distanced or distal nursing. This approach draws on recent work in nursing (Foster et al., 2006; Muncey, 2005; Peterson, 2015) as well as older writers such as Lawler (1991) and Sandelowski (1994). Peterson (2015: 229) cites (Frank, 2000) to remind us that ‘by telling stories of illness, authors can bridge the divide between lived experience of illness and academic analysis of those experiences’. My experiences in hospital gave me exactly that opportunity. Even as I was admitted to hospital, and then to high dependency in a local hospital in the South East of England with COVID symptoms, my prior experiences as a nurse were in my thoughts as I interpreted my experiences as a patient. As nurses explained the need to have CPAP, I remembered and drew on knowledge from nursing patients on CPAP as a clinician to cope with my anxiety. As I noted in hospital, my reflexivity as an ethnographer couldn’t be left behind even as I gasped for breath.

The key method I used was participative observation (Jackson, 1989; Savage, 2000) and detailed note taking both in hospital and after discharge in ‘a continuous and deepening interpretive process’ (Van Maanen, 1988: 18). Thematic analysis was used to analyse data from my field notes.

## Ethical considerations

Issues related to consent and anonymity (Morse, 2002) and balancing goods and harms must be considered by all researchers. No consent to use the data was required as the story is my own; I use my experience without describing the details of staff who cared for me and I focus on the particular to comment on the general (Pariseau-Legault, 2018). In autoethnography, one needs to answer the question: what good will my story do? The purpose of this article is to understand the process and experience of nursing in the pandemic from a patient perspective. As a patient who is fundamentally empathetic to nursing and also as a nurse, nurse teacher and researcher, I seek to understand and theorise the experience of nursing and social context which shapes both nursing and the patient experience (Pariseau-Legault, 2018: 39). Another way to validate an autoethnography is to reflect that qualitative research findings illuminate human experience and should be meaningful and transferable (Guba and Lincoln, 1989).

## Findings – reflections on my patient experience

I contracted COVID 19 in March 2020 and was treated at home by my community doctor for 6 weeks. I deteriorated and was admitted as an inpatient for 10 days. My main symptoms were pyrexia, breathlessness, low oxygen saturation levels and extreme fatigue. I was started on CPAP 8 hours after my admission to emergency care and remained on CPAP for 20 hours. I was nursed in a single isolation room. During the acute stage of my illness, the priority of the nurses was to monitor my status and prevent deterioration. Qualified nurses were assiduous in their recording of my observations and I felt safe under their observation. As my condition stabilised, the observations were undertaken by HCAs, the data directly fed into an iPad and monitored by the ward manager for the shift who would have been alerted if my data had been abnormal. At this stage, I had less access to registered nurses apart from when they came into my room to administer my medications and as a result, I began to feel less safe. I saved up my questions for those short moments when I knew the nurse looking after me would be around: at the beginning of the shift when they assessed my condition and their workload; and towards the end of the shift when they wrote a report for the shift. HCAs made my bed, took my observations, brought me in my meals and frequent cups of tea; yet seemed reluctant to answer my questions and frequently deferred them to the ‘nurse looking after me’.

I received excellent care in many respects. Many of the staff, cleaners, catering staff, nurses, HCAs and medical staff, were kind and efficient. Examples include the HCA who bought me some lacto-free milk from the hospital shoppe; the cleaner who went out of his way to chat to me while he cleaned my room; the emergency doctor who sought to find the underlying cause of my falling oxygen saturation levels and explained everything as he did so. I focus on three findings from my experiences of being a distanced patient during the COVID pandemic: intimate nursing care, communication and patient experience.

### Intimate care in isolation

During the initial stages of my admission, I was not offered a wash or to have my teeth cleaned. I was hydrated intravenously and was encouraged when possible by the physiotherapists to drink as much water as I could. I went to the toilet in breaks from CPAP assisted by the nurses who carefully removed the equipment; my toilet visit allowed me to wash my hands in the hand basin in my single room. Once I began to feel better as my oxygen saturation levels began to rise and I stabilised off CPAP, I had more energy and asked for a wash. My room had a toilet with hand basin and a second hand basin in the main room. I was able to manage a basin wash and a change of underwear. During my stay in this single room, I assumed that the notice above the taps saying ‘cold’ meant that there was no hot water. I therefore washed with cold water for 2 days until I commented on the lack of hot water to a HCA. She laughed and explained that only applied to the toilet hand basin. None of the nurses asked me if I needed help with washing or explained that I would not be able to use the usual shared bathroom and shower facilities. Towards the end of my stay, I managed to wash my hair in the hand basin after asking for a jug. The nurses and the HCAs, were assiduous in making my bed with fresh sheets every morning.

### Communication as an isolated patient

When I was critically ill, I felt fully informed about what my treatment plan was likely to be, what options might be considered next and what progress was being made. As my condition improved, I felt less well informed of decisions which were made regarding discharge and treatment plans. Medical staff seemed to make the decisions outside my room on the ward round and then amended them outside my room after seeing me. Their plans were communicated to the nursing staff who then communicated them to me. Any comments I had on the plan of treatment or discharge was conveyed back through the nursing team to the medical staff to be discussed at the next morning’s round. As a result of this distanced, lengthy communication processes, I wrote down questions for the nursing and medical teams to ensure I did not forget them. This caused me further distress as I did not want to forget them. At no stage did anyone ask about my home circumstances and my husband received no communication from the hospital for the entire time I was an inpatient; it was as if my home life was a separate existence. I found the isolation difficult and missed visits from my family. I reflected in my notes how quickly this aspect of nursing care, the care of relatives, had disappeared from nursing. Only once when I began to come off CPAP, did the medical team offer to inform my husband of what was happening. I told them that I was in contact with home. However, none of the nurses enquired about my home circumstances or asked on admission, or at any point during my stay or on discharge if my family needed to be informed or what my home circumstances were.

My notes record that I used the soft technologies at my disposal. I adapted the single room space, keeping my blinds up to see staff outside during the day, using my mobile telephone to communicate with family and friends as ways to combat isolation. I also adapted my communication by using hand signals, miming a need for fluids for example, to attract staff attention through the closed doors. I smiled a lot and enquired about HCAs’ circumstances in order to retain them in my room.

### The patient experience – empathy and feeling cared for

Empathy expressed by staff for my experience as a patient was more evident in emergency care and while I was critically ill on the high dependency COVID ward. While on CPAP, I felt safe and able to communicate with the nurses who responded to my needs with hand signals and written notes. They tolerated my fear and along with my own familiarity with CPAP, I felt that their care helped me to respond to CPAP well.

After I came off CPAP, I was transferred to another ward but I remained breathless. I was diagnosed with underlying bronchiectasis. Once my admitting observations were noted, I was left with the patient bell and told to call if I needed to. I have never felt so alone and scared; it was an effort to breathe and I felt isolated in my single room connected to the staff only by a bell and unable to see any nursing staff. I sent a text to a friend that I was frightened and scared I would die. I was struck by the lack of empathy from the ward nurse and the HCA who accepted my transfer to the new ward. They did not acknowledge my anxiety; perhaps they were unaware of how scary breathlessness can be? Perhaps they were used to seeing breathless patients or found my distress too distressing? Despite nebulisers during the night, it was only when I called the nurse and said forcefully that I felt abandoned and scared, that she called the doctor and I was written up for medication which relaxed my airways, enabling me to rest. The nurse on a morning shift, when I was again breathless and wheezy, was able to relax me by saying quite simply, ‘You poor thing. You don’t know how to get comfortable do you?’ She touched my head and stayed with me for longer than it took her to complete my observations. A night nurse was also kind to me during periods of breathlessness. In general, then, the empathy I felt from the ward nurses varied over the course of my stay in hospital. For example, when I told a ward manager that I was worried about going home and becoming breathless again, she replied that being breathless was no reason to keep me in hospital and that I’d have to learn to cope with my symptoms at home. In contrast, meeting the respiratory nurses who managed my discharge was a different experience. I felt they empathised and I felt safe in their care even though I was going home and would only have telephone contact with them.

## Discussion

The findings illustrate that nurses delegated care to HCAs once my position stabilised; this delegated care focussed on observations and bed-making (technical) care rather than relational (affective) care. My patient experience was distressing and nurses’ and HCAs’ responses to my distress varied. There was very little attention paid to intimate (basic) care or to non-essential communication (affective care). Where communication was necessary, explaining my situation in emergency care or when I was connected to CPAP, it was effective. Communication was driven by consideration of tasks such as medications, observations or discharge. Opportunities for informal conversation were reduced to the cleaning staff or as HCAs made my bed. Questions about my care made to HCAs were directed to nurses who were responsible for technical care. As nurses were not involved with technical or affective care during the later stages of my hospital admission, this process lengthened the communication process. The findings also illustrate the disappearance of the family from nursing care and the transfer of this task to medical staff.

The isolation nursing I experienced constructed a distanced form of caring. I became a distal patient who adapted artefacts and soft technology to survive. I argue that this form of care was possible because NPM constructs commodified nursing where the subjective experience is removed from the nurse-patient relationship. I argue that the emergence of commodified care has only been possible because it is grounded in historical forms of nursing which devalue relational and intimate care in preference for technical care. In some ways, this new form of commodified care has been waiting to happen since the emergence of NPM.

However there might have been something else at play in the nurses’ responses to my situation. I draw on Dashtipour et al.’s (2021) writing on psychosocial thinking to discuss the forms of distanced care I have described to argue that they might have been underpinned by the anxiety experienced by the nurses in the pandemic generally, and in the face of my symptoms in particular, that is my breathlessness.

### Anxiety and avoidance

I found my admission and symptoms, particularly the breathlessness, overwhelming. My anxiety was tolerated by some nurses particularly when I was critically ill when they were able to manage their own anxiety in order to care for me (Allan, 2011; Fabricius, 1991). With others, I experienced a lack of understanding and empathy for my symptoms, particularly when I was improving and (in their words) ‘no longer needed to be in hospital’. These experiences are not unusual in patients who have been nursed in isolation (Barratt et al., 2010; Purssell et al., 2020). Isolation nursing prevents normal interpersonal relationships and restricts communication; it influences the quality of care and opportunity for emotional support of the patient. Patients can feel anxious, angry, frustrated and fearful especially if communication is poor (Barratt et al., 2010).

In my case, I suspect that the nurses felt alienated from the distanced form of care they were constrained to give (De Kock et al., 2021; Downar and Kekewich, 2021; Swaminathan et al., 2021) despite the lack of value they might have assigned to intimate care or even to observations in a non-critical patient. Drawing on Menzies-Lyth’s 1960 thesis, I argue that the nurses’ seeming lack of connectedness and empathy was shaped by the defences against anxiety caused by patients’ distress over their illness. Another reading of their response to anxiety is helplessness which health care staff have reported feeling during the pandemic (Hu et al., 2020; Unison, 2021). Wentzel and Brysiewicz (2014) and Lappeman and Swartz (2020) report nurses’ distress at witnessing patients’ pain and their helplessness at being unable to relieve it. Ways of managing their personal distress are to avoid contact with patients who are in pain or distress, and to act defensively to protect themselves (Frenkel, 2002; Lappeman and Swartz, 2020). In their study, Lappeman and Swartz (2020) interpreted nurses’ defencive behaviour and avoidance of relational caring in the face of patients’ pain and distress as nurses not wishing to acknowledge pain if it were culturally unacceptable to express pain. My distress and expression of that distress might also have been interpreted as culturally inappropriate. As Dashtipour et al. (2021) argue, these intrapersonal factors must be understood and examined within the context that care is delivered; the context shapes and constrains nurses’ ability to provide relational care (Smith and Mackintosh, 2007).

### Distanced or distal nursing

My experiences of isolation nursing suggest that the focus on infection control, isolation, the technical aspects of my care, produced a form of distal care where I felt distanced from the nurses caring for me. May et al. (2006) showed the emergence of the distal patient in e-health technologies and Allan et al. (2009) showed how e-health in fertility clinics has constructed a distal patient pathway. These data show a distal form of nursing in hospital work. The hard, material artefacts and the soft technology constructed a form of distal patient (May et al., 2006). The reliance on exaggerated modes of communicating such as gestures, facial expressions, proxemics (use of space in communication), body language, touch and intensity of gaze a new form of communication all served to alienate me from my carers (Senicola et al., 2020). The lack of my family added to my sense of disconnectedness from both the ward and the world outside the hospital. Isolation nursing in single rooms created a new use of space where patients were cut off from other patients and from their assigned nurse. The ward was not a public space as patients were restricted to their rooms and families and friends prohibited from visiting.

### Commodification of nursing care

In the pandemic, new forms of nurse-patient relationships mediated through new material artefacts have emerged; in creating new artefacts to deliver nursing, new patients are also created. The distal patient, who relies on different modes of communication compared with traditional face-to-face communication in hands on care, uses signing, artefacts and relies on his/her own inner resources because of reduced contact. These new forms of communication do not rely on the individual nurse–patient relationships but on the collective nature of routines and task allocation (Allan et al., 2008) along with the delegation of lower value nursing tasks to HCAs. The new distal patient is a product of trends in nursing work organisation which are older than the pandemic. They build on nurses’ tendency to avoid relational care as they struggle to deal with the anxiety of care. These forms of distal nursing are shaped by a tradition of not providing and valuing relational care in nursing, along with the structural reorganisation of nursing where the provision of relational and intimate care along with lower value tasks are delegated to HCAs. This is part of a continued commodification of nursing care in the British NHS (Dashtipour et al., 2021; Hyde and Davies, 2004). In the commodification of nursing care, tasks can be broken down against skill, workload, patient need and characteristics, treatment characteristics, patient turnover, nurse-patient ratios or task-level, job-level and unit-level workloads (van Den Oetelaar et al., 2016). Whatever measure is used to predict and control nursing work, the emphasis is predict and control rather than individual professional decision making. Removing the patient’s subjectivity and his/her family or social context in the distal form of caring which has emerged in hospital nursing during the pandemic, NPM has achieved what has been contested and resisted over the years, a form of nursing which is commodified and controlled rather than responsive to patient need. This has been possible because of traditional tendency to avoid relational care and value technical care in nursing as a defence against the anxiety of care.

## Concluding thoughts

Forms of distanced caring were introduced during the pandemic as health care providers sought to protect their staff from contact with patients in order to reduce the risk of spreading COVID-19. But this occurred within the context in the UK at least, of the division of nursing work into higher and lower level tasks allocated on the basis of ‘complexity’ to either registered nurses or unregistered HCAs. Within NPM, all nursing tasks are managed by algorithm by ward managers who have very little contact with individual patients. I believe that this new distanced form of nursing has been possible partly because of the higher value nurses have always placed on technical care against intimate nursing care. And partly because of nursing’s lower status as a health profession which has been particularly evident during the 2020 COVID pandemic but has actually built on nursing’s position with a structurally unequal health system which nurses in leadership positions have been reluctant to criticise.

We are yet to learn the full effects on nursing staff of witnessing such appalling death, acute and severe illness during a pandemic and nursing in this distanced way. We do not yet understand the ways in which distanced nursing shapes their ability to manage, respond and tolerate patients’ anxiety and respond in a humane and empathic way. The pandemic again raises important questions about the nature of nursing which have continued to dog nurses and nursing for some decades. The fact that some nurses successfully negotiated and resisted distanced forms of caring and managed to hold on to holism in the midst of the pandemic is perhaps a positive sign that such constraints on nursing can be thought about and resisted.
